# Competitive mapping allows for the identification and exclusion of human DNA contamination in ancient faunal genomic datasets

**DOI:** 10.1186/s12864-020-07229-y

**Published:** 2020-11-30

**Authors:** Tatiana R. Feuerborn, Eleftheria Palkopoulou, Tom van der Valk, Johanna von Seth, Arielle R. Munters, Patrícia Pečnerová, Marianne Dehasque, Irene Ureña, Erik Ersmark, Vendela Kempe Lagerholm, Maja Krzewińska, Ricardo Rodríguez-Varela, Anders Götherström, Love Dalén, David Díez-del-Molino

**Affiliations:** 1 Globe Institute, University of Copenhagen, Copenhagen, Denmark; 2 Archaeological Research Laboratory, Department of Archaeology and Classical Studies, Stockholm University, Stockholm, Sweden; 3 Department of Bioinformatics and Genetics, Swedish Museum of Natural History, Stockholm, Sweden; 4 Centre for Palaeogenetics, Stockholm, Sweden; 5 Department of Zoology, Stockholm University, Stockholm, Sweden; 6 Department of Organismal Biology, Human Evolution, Uppsala University, Uppsala, Sweden; 7 Department of Biology, University of Copenhagen, Copenhagen, Denmark; 8 Department of Animal Breeding, INIA, Madrid, Spain

**Keywords:** Ancient DNA, DNA contamination removal, Palaeogenomics, Competitive mapping

## Abstract

**Background:**

After over a decade of developments in field collection, laboratory methods and advances in high-throughput sequencing, contamination remains a key issue in ancient DNA research. Currently, human and microbial contaminant DNA still impose challenges on cost-effective sequencing and accurate interpretation of ancient DNA data.

**Results:**

Here we investigate whether human contaminating DNA can be found in ancient faunal sequencing datasets. We identify variable levels of human contamination, which persists even after the sequence reads have been mapped to the faunal reference genomes. This contamination has the potential to affect a range of downstream analyses.

**Conclusions:**

We propose a fast and simple method, based on competitive mapping, which allows identifying and removing human contamination from ancient faunal DNA datasets with limited losses of true ancient data. This method could represent an important tool for the ancient DNA field.

## Background

Right after the death of an organism, microbial communities colonize the decomposing tissues and together with enzymes from the organism they start degrading the DNA molecules [1–3]. DNA degradation is dependent on time and environmental variables such as temperature but also humidity and acidity [4]. Even though the specific model for DNA decay is still debated and it is likely multifactorial [4], the consequence is that ancient remains typically contain very few molecules of endogenous DNA and these sequences are characterized by short fragment sizes [5].

A second major challenge of ancient DNA research is contamination from exogenous sources [6, 7]. Environmental DNA molecules in the soil matrix the ancient sample was recovered from can easily overwhelm the small amounts of endogenous DNA. This is also true for DNA from people who collected and handled the samples in the field and/or museum collections [8, 9]. While the use of Polymerase Chain Reaction (PCR) technology allowed ancient DNA research to overcome low concentration problems, the sensitivity of the PCR has made it very difficult to avoid introducing modern contaminant sequences among the authentic ancient DNA [10].

In the last decade, together with more refined DNA extraction and laboratory methods tailored to efficiently retrieve very short and scarce DNA sequences [5, 11], it has become possible to obtain massive amounts of sequences from ancient material using high-throughput sequencing technologies. These technologies have allowed the recovery of hundreds of ancient human (reviewed in [12]) and other high quality ancient faunal genomes such as those from horses [13], wooly mammoths [14], and bears [15]. However, the challenges from exogenous contamination remain and have sparked a search for computational methods to identify and monitor contaminant DNA sequences in ancient sequencing datasets.

Aside from the short fragment size, the other most notable characteristic of ancient DNA is post-mortem damage. After death, the repairing mechanisms of DNA damage such as hydrolysis and oxidation stop functioning, and this damage accumulates in predictable patterns [16, 17] The most common ancient DNA damage is deamination of cytosines to uracils in the overhangs of fragmented DNA molecules [16, 18, 19]. This results in an excess of C to T substitutions in the 5′ end (and G to A in the 3′ end) of ancient DNA sequences. Since this feature is very common in sequences derived from ancient DNA sources and absent in younger samples, it has been widely used as a key criteria to authenticate ancient DNA experiments [5, 20].

In modern-day ancient DNA studies, exogenous sequences are differentiated from real ancient sequences from the source organism by mapping all sequences to a reference genome and keeping only those that result in alignments with less than a defined number of differences [21, 22]. This approach to circumvent environmental contamination has gained general acceptance, and currently exogenous contaminants are at most considered problematic due to their consumption of sequencing capacity. However, the probability of spurious alignments from exogenous sequences occurring by chance increases with decreasing sequence length [23]. In order to avoid these, thresholds for minimum fragment length, that still allow for enough specificity of the alignments, are used [24–26].

Modern human contamination is especially problematic for human palaeogenomic studies since ancient, anatomically modern humans typically fall within the variation of modern humans [27, 28]. This has led to the development of a plethora of methods aimed at computationally quantifying and monitoring exogenous contamination in ancient human DNA datasets [29]. However, the number of methods that allow for the effective exclusion of this type of contamination remains limited. For example, Skoglund et al. [30] used the differential empirical distributions of post-mortem damage (PMD) scores, based on both base quality scores and their level of polymorphism with respect to the reference genome, to differentiate DNA sequences from ancient and modern samples. The PMD scores in a contaminated ancient sample could then be used to successfully identify and separate the sequences that are most likely to have originated from an ancient template molecule from the contaminant ones. Even though this method can allow for the enrichment of the proportion of ancient sequences several-fold in respect to the contaminant sequences, the amount of data lost in the process is very large (45–90%) depending on the age of the ancient sample [30].

Here we use competitive mapping to investigate the presence of exogenous sequences in ancient sequencing files to evaluate the pervasiveness of human contamination in ancient faunal DNA studies. Previous ancient DNA studies have used similar strategies, i.e. mapping the sequenced ancient DNA data to several reference sequences at the same time, to identify target microbial species (e.g. [31, 32]). We use competitive mapping to identify the levels of contamination in ancient faunal sequencing files and characterize the exogenous sequences by using summary statistics to compare them to those of authentic ancient DNA. We then present this strategy as a simple and fast method that enables the conservative removal of human contamination from ancient faunal datasets with a limited loss of true ancient DNA sequences.

## Results

**Fig. 1:**
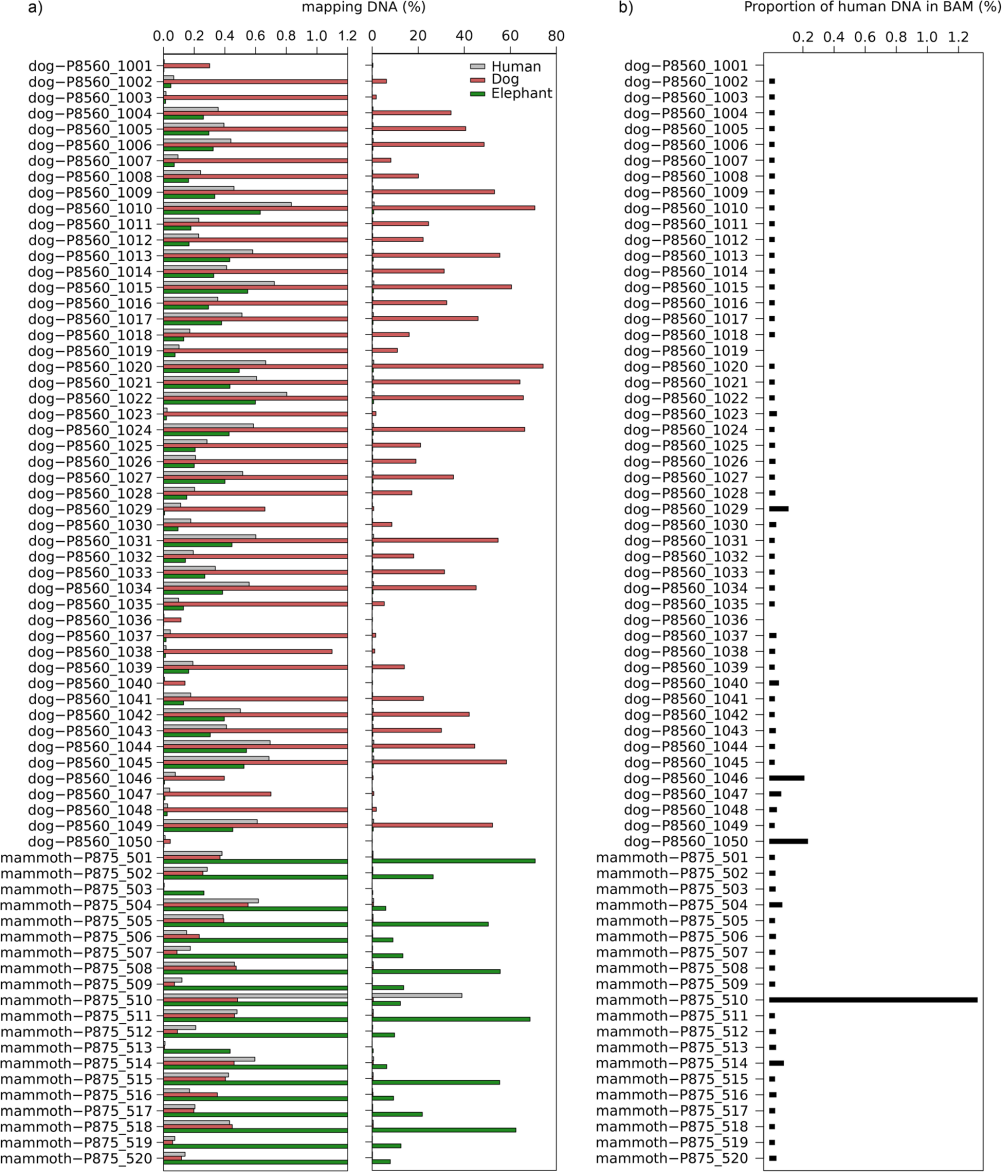
Mapping statistics for target, non-target and human references. **a** Right panel, percentage of reads from each sample mapping to each of the three reference genomes. Left panel, same as before but zoomed to percentages below 1.2%. **b** Proportion of reads from the faunal BAM file that mapped to the human part of the concatenated reference genome

We first mapped the raw reads from all sequenced ancient samples (50 dogs, *Canis lupus familiaris*, and 20 woolly mammoths, *Mammuthus primigenius*) to three separate reference genomes: the African savannah elephant, dog and human. We found variable levels of sequences confidently mapped to foreign reference genomes (average 0.25% for non-target and 0.86% human) in these sequencing files (Fig. 1a). Most of the files (> 95%) contained less than 0.071% of sequences mapped to human and 0.054% the non-target species. We then estimated average read length (mRL) and post-mortem damage scores (PMD^R^) for all alignments. We detected some significant differences in these indices between sequences mapping to target and to non-target and human (Fig. S1). However, most comparisons between the sequences mapping to the non-target species and human references were not significant.

**Fig. 2:**
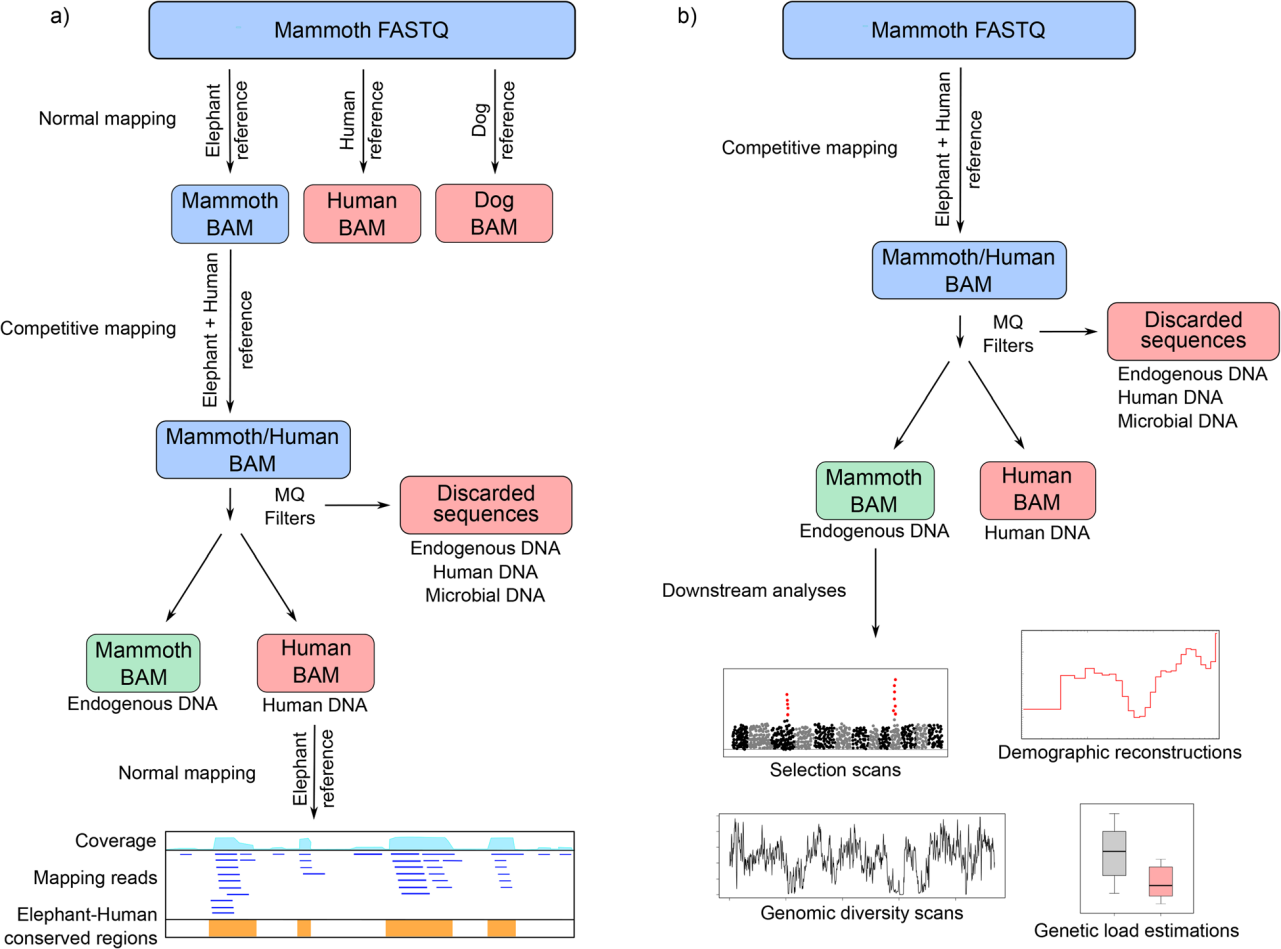
Schematic view of the competitive mapping analyses. FASTQ files represent ‘raw’ sequencing files and BAM files represent alignments to a reference genome. Color boxes indicate different types of data: blue, files that need further processing; red, discarded data; and green, data for downstream analyses. **a** Schematic view of the analyses performed in this manuscript. An example using a mammoth sample is shown. First, normal mapping to the elephant, human and dog references to check for endogenous content as well as non-target and human contamination in the sequencing files. Second, competitive mapping to a concatenated reference of an elephant and human to detect human contamination in the alignments. Third, normal mapping human data to the elephant reference to check that the human contaminat sequences map preferentially to conserved regions of the genome. **b** Schematic view of a typical competitive mapping pipeline using a mammoth sample as example. After competitive mapping, only the sequences mapping to the elephant part of the concatenated reference will be used for downstream analyses

To investigate whether the target BAM files contain human contaminant sequences we remapped the aligned reads to a concatenated reference composed by the reference genome of the target species, dog or elephant, and the human reference genome (Fig. 2a). The concatenated reference was created by merging the two relevant reference genomes together to create one fasta file containing all chromosomes for each species. This *competitive mapping* approach allowed us to differentiate between three kinds of reads contained in the target species BAM files. First, reads which align to the target reference genome and not to the human reference genome. These sequences represent the endogenous alignments that originate from the sample and not from human or microbial contamination. Second, reads which align to the human reference genome and not to the target species reference genome. These sequences represent the fraction of human contamination in the faunal BAM files. And third, reads that align to both the target reference and the human reference genomes. These sequences could have three origins, 1) true endogenous sequences from regions of the genome highly conserved or identical to the human genome, 2) human contaminant sequences from regions of the genome highly conserved or identical to the target genome, or 3) microbial contaminant sequences that would align to any mammalian genome by random chance. In any case, because these sequences map to both target and human reference genomes at the same time they would thus be discarded when applying mapping quality filters (Fig. 2a).

**Fig. 3:**
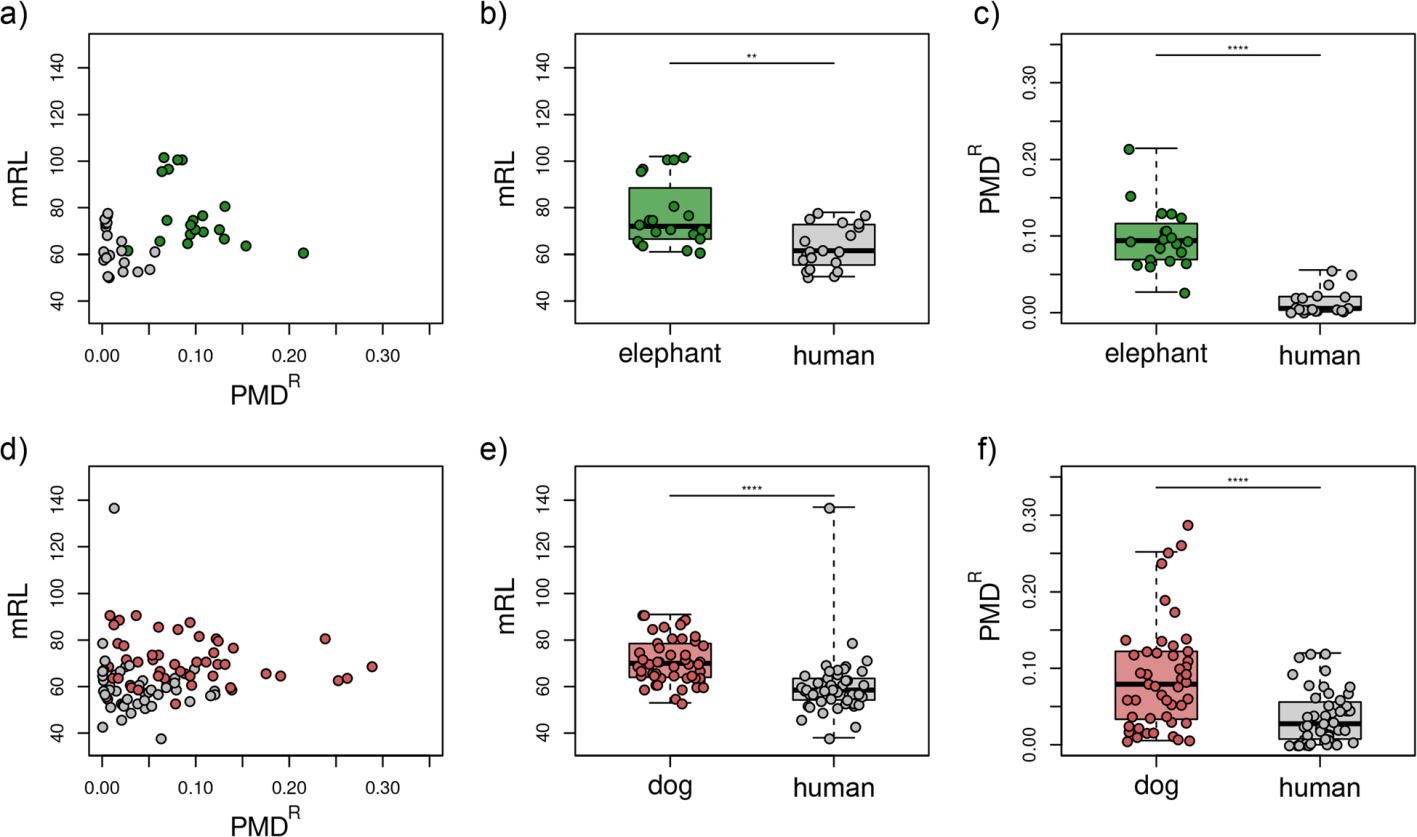
Characterization of endogenous and human contaminant reads in faunal BAM files. **a** Comparisons of PMD^R^ and mRL for all mammoth samples. **b** mRL for mammoth sequences mapping to the elephant or the human parts of the concatenated reference (Wilcoxon rank-sum test, W = 313.5, *p*-value = 0.00223). **c** PMD^R^ for mammoth sequences mapping to the elephant or the human parts of the concatenated reference (Wilcoxon rank-sum test, W = 397, *p*-value = 1.016e-10). **d** Comparisons of PMD^R^ and mRL for all ancient dog samples. **e** mRL for dog sequences mapping to the dog or the human parts of the concatenated reference (Wilcoxon rank-sum test, W = 1929, *p*-value = 1.251e-08). **f** PMD^R^ for dog sequences mapping to the dog or the human parts of the concatenated reference (Wilcoxon rank-sum test, W = 1743, *p*-value = 1.511e-05). In all cases, **: *p*-value < 0.01 and ****: *p*-value < 0.0001

For each sample, we extracted the reads aligned to the target species of the concatenated reference, representing the true ancient sequences, as well as the human, representing the amount of human contamination contained in the original target BAM file. We found that the alignment files from almost all samples contained sequencing reads that preferentially mapped to the human part of the reference genome than to the target part (average 0.03%; range 0–1.3%) (Fig. 1a, Supplementary Table 1). However, we caution that, because an unknown fraction of the reads discarded due to the mapping quality filters should also be human contaminant, the fraction of reads in the human part of the concatenated reference represents only a lower bound for the amount of contamination in the original faunal BAM file. Finally, both mRL and PMD^R^ were significantly lower in the sequences mapped to the human part than in the ones mapped to the target (Fig. 3).

**Fig. 4:**
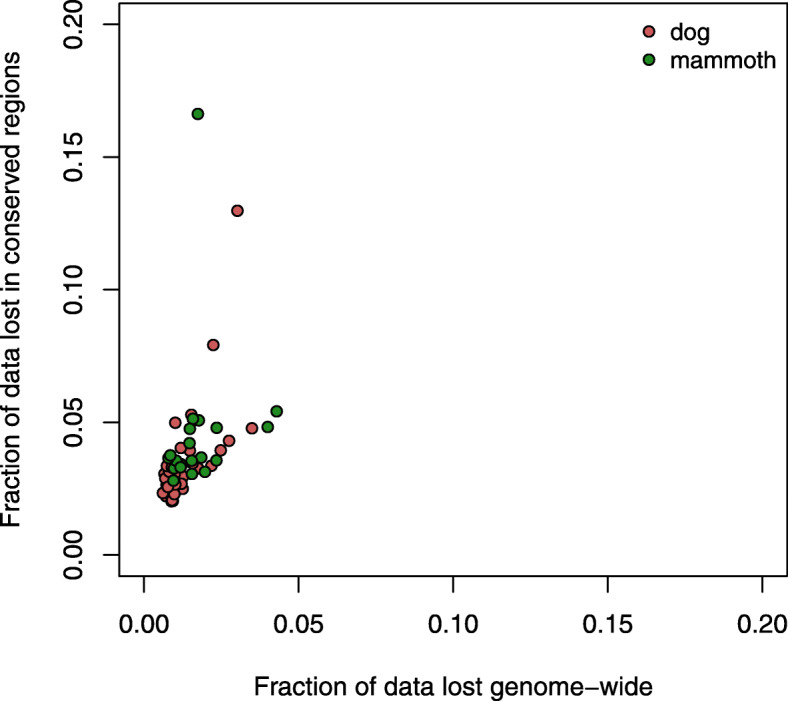
Data lost per sample after competitive mapping. Fraction of data lost in each sample at genome-wide level and only in conserved regions. Colors indicate different species

When using competitive mapping, a fraction of sequences that align to both the target and the human parts of the concatenated reference, were lost (Fig. 2a). Our results indicated that this fraction was an average of 1.33% of the total number of reads per sample (range 0.6–4.3%, Fig. 4, Supplementary Table 1). However, when accounting only for conserved regions between the target species genome and the human genome, the amount of lost sequences was higher (average 3.65%; range 2–16.6%).

## Discussion

### Contamination in raw sequencing files

**Fig. 5:**
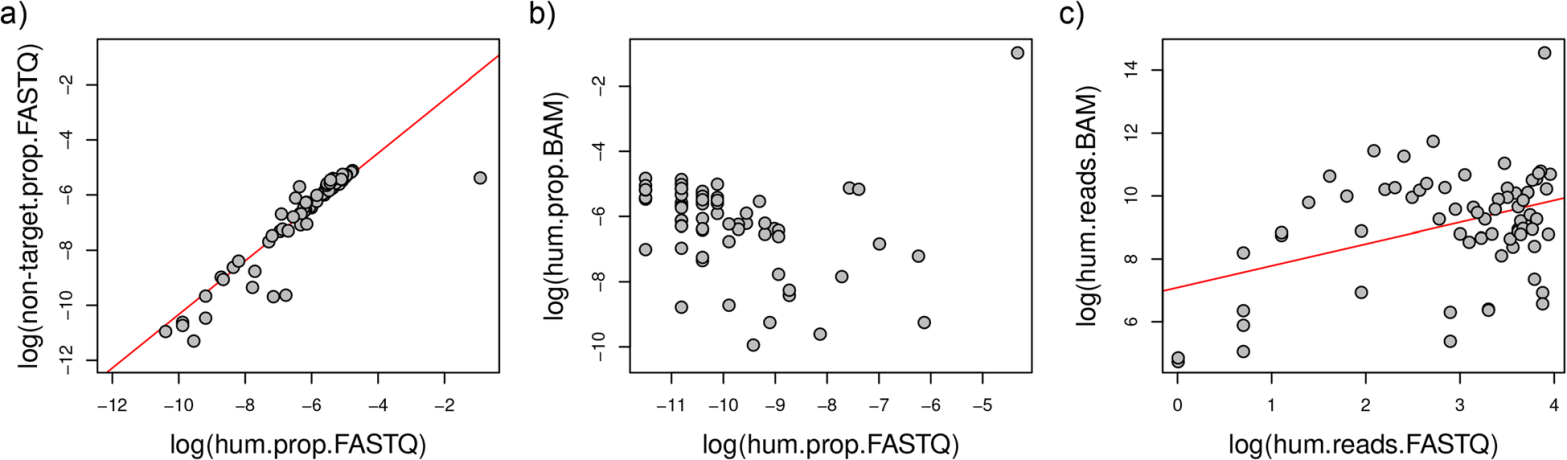
Proportions of sequences mapping to human, target and non-target reference from the FASTQ and BAM files. **a** Correlation between the proportion of reads mapping to human and to the non-target species in the raw FASTQ sequencing files (r^2^ = 0.81, F = 303.8, *p*-value = < 2.2e-16). **b** Not correlation between the proportion of reads mapping to human in the raw FASTQ sequencing files and the proportion of reads mapping to human from the faunal BAM file (r^2^ = 0.01, F = 1.67, *p*-value = 0.2). **c** Correlation between the number of reads mapping to human in the raw FASTQ sequencing files and the number of reads mapping to human from the faunal BAM file (r^2^ = 0.15, F = 13.5, *p*-value = < 2e-16)

Overall, we found low levels of sequences mapped to foreign reference genomes in the raw sequencing files (Fig. 1a). The proportion of reads mapping to the non-target species and human for each sample were highly correlated (Fig. 5a), indicating that they mostly represent sequences from the target species that map to conserved regions in the other two reference genomes. However, there were notable outliers in the amount of faunal sequences mapping to the human reference. For example, one sample contained a higher proportion of sequences mapped to the human (38.9%) than to the target species (12.3%). This suggested that there could be high levels of human DNA contamination in particular sequencing files.

When characterizing mRL and PMD^R^ in the sequences mapping to the different reference genomes we found some differences between the sequences mapping to target compared to non-target and human (Fig. S1), in line with the latter being mostly composed by shorter sequences mapping to conserved regions and the former mostly true endogenous reads. In fact, our results suggest almost no differences between the sequences mapping to the non-target species and human references, reinforcing the idea that these two files are composed of sequences with a common origin.

### Human contamination in faunal BAM files

Given that we detected contaminant human sequences in all our ancient fauna sequencing files, we next used competitive mapping to explore whether these contaminant reads can be also found in the BAM file of the target species that would be used for downstream genomic analyses. We found that the BAM files from almost all samples contained sequencing reads that preferentially mapped to the human part of the concatenated reference genome, but the proportion was generally low (Fig. 1b). Interestingly, the proportion of reads mapped to the human reference from the raw data and the fraction of reads mapping to the human part of the concatenated reference in the target BAM after competitive mapping are not correlated (Fig. 5b). The reason for this is that the proportion of human reads in the BAM file also depends on the endogenous content of each sample. In fact, the total amount of human sequences that make it to the BAM files is proportional to the number of human sequences in the FASTQ (Fig. 5c). This indicates that the amount of human contamination that is retained in the target BAM files after alignment to the target reference genome can be roughly predicted from the amount of human contamination in the raw sequencing files.

We then estimated mRL and PMD^R^ for the true ancient sequences and the contaminant sequences. For both mammoth and dog samples we found a clear distinction in PMD^R^ of the sequences mapping to the target species and the ones mapped to human, with higher PMD^R^ for the target species, representing true ancient sequences, and lower for the human sequences (Fig. 3c, f). However, we found that the contaminant human reads also displayed a lower mRL (Fig. 3b, e). This was contrary to the expectation of modern human contaminant sequences being longer than true ancient sequences, but can be explained by the fact that shorter contaminant sequences align easier to evolutionary conserved regions of the target species reference genome than longer sequences [26, 33].

### Excluding contaminant reads from faunal BAM files

The presence of contaminant human sequences in ancient faunal BAM files can be challenging for any downstream analyses that are based on evolutionary conserved parts of the genome, such as coding regions, since the contaminant sequences are concentrated in these regions. Other downstream analyses based on genome-wide scans such as estimations of heterozygosity, estimation of inbreeding levels using runs-of-homozygosity, or analyses focused on the presence of rare variants [34] can be highly affected by the emergence of false variants caused by human contamination [35, 36]. This is especially true for analyses based on low to medium coverage samples, such as most ancient DNA studies. Additionally, since an unknown fraction of the reads discarded using competitive mapping can be of human origin, our detected levels of exogenous human sequences in ancient faunal alignments represent only the lower bound of contamination for these files.

We therefore propose that the method applied here, using competitive mapping of the raw data to a concatenated reference genome composed by the reference genome of the target species and the human genome, represents a fast and simple approach to effectively exclude contaminating human DNA from ancient faunal BAM files (Fig. 2b). An additional advantage of this approach is that a portion of contamination from short microbial reads, common in ancient datasets [26], should also be excluded with this method as many of these short reads would align to both target and human parts of the concatenated reference and are filtered out using the mapping quality filters.

One relevant downside of using competitive mapping could be the loss of data. True ancient sequences from the target species that belong to conserved regions of the genome and are identical between the target species and human, would align to both parts of the concatenated reference, and thus be lost when using the mapping quality filters. However, our results indicate that the amount of data lost this way is very limited in a genome-wide context (average 1.3%), and slightly concentrated in conserved regions of the genome (average 3.65%). Unfortunately, we do not have a practical way to estimate what fraction of those sequences are true target sequences and how many are of human or microbial origin.

## Conclusions

We show that variable levels of contaminant human sequences exist in ancient faunal datasets. To some extent, this human contamination persists even after sequence reads have been mapped to faunal reference genomes, and is then characterized by short fragment lengths that are concentrated in evolutionary conserved regions of the genome. This results in human contaminant sequences being included in ancient faunal alignment files and thus have the potential to affect a range of downstream analyses. To address this, we here propose a fast and simple strategy: competitive mapping of raw sequencing data to a concatenated reference composed of the target species genome and a human genome, where only the sequences aligned to the target part of the concatenated reference genome are kept for downstream analyses. This approach leads to a small loss of data, but allows for the effective removal of the putative human contaminant sequences.

Contamination is a key issue in ancient DNA studies. Preventive measures both during field collection and in the laboratory therefore remain a critical aspect of ancient DNA research [36, 37]. There is a growing array of computational methods that allow to confidently identify contamination levels (reviewed in [29]), but few that allow to efficiently separate authentic ancient sequences from contaminating DNA [26, 30]. Thus, the method we propose here represents an important addition to the selection of tools aimed at computationally reducing the effects of human contamination in ancient faunal DNA research.

## Methods

### Materials

We analyzed genomic data from 70 ancient and historical mammalian specimens, 50 dogs and 20 woolly mammoths (Supplementary Table 1). The materials derived from dogs originate from a variety of contexts (ethnographic collections and archaeological excavations) and materials (teeth and bones) which have been stored in museum collections for up to 125 years after collection/excavation. The twenty mammoth samples were all collected in Wrangel Island in several expeditions along the last 30 years and are radiocarbon dated.

### Laboratory procedures

For all samples, the outer layers of bones, teeth and tusk were removed using an electric powered drill (Dremel, USA) in order to minimize external contamination. Approximately 50 mg of bone powder was recovered from inside the bone, tooth or tusk using an electric drill operated at low speed. We then extracted DNA from all samples using the silica-based protocol described in Ersmark et al. [38]. Thirty-four of the dog samples were additionally subjected to a pre-digestion step, incubated with EDTA, urea, and proteinase K for one hour at 55 °C, to further reduce the amount of contamination within the extract by removing the superficial DNA. We did not treat any of the extracts with USER enzyme in order to enable assessment of post-mortem damage rates following DNA sequencing.

We constructed Illumina genomic libraries for sequencing from the DNA extracts using established ancient DNA protocols [39, 40]. All libraries were amplified using indexes unique for each sample and were subsequently pooled and sequenced on a total of 4 lanes on the Illumina HiSeq2500 platform at the National Genomics Infrastructure (Science for Life Laboratory, Stockholm), using paired-end 2x150bp settings.

### Data analyses

We trimmed sequencing adapters and merged paired-end reads using *SeqPrep v.1.1* (github.com/jstjohn/SeqPrep) with default settings (excluding sequences shorter than 30 bp after merging) and a slight modification of the source code to calculate the base qualities in the overlapping region [14]. We then mapped the merged reads to three separate reference genomes: the African savannah elephant genome (LoxAfr4, Broad Institute), the dog genome (CanFam3.1, [41]), and the human reference genome (Hg19). All mappings were performed using *BWA aln v0.7.8* [42] using settings adapted for ancient DNA as in Pečnerová et al. [43].

We removed PCR duplicates from the alignments using a script (github.com/pontussk/samremovedup) that takes into account both starting and end coordinates of the reads to be identified as duplicates [44] and estimated the number of unique mapping reads using *samtools v1.8* [45]. In all cases, we refer to *mapped reads* to those sequences retained after filtering by mapping quality > 30. We consider true endogenous sequences are those mapping to the target species (i.e dog reference for ancient dog samples and elephant reference for mammoth samples) and exogenous contaminant sequences are those mapping to the non-target reference (i.e elephant and human references for ancient dog samples and dog and human references for mammoth samples). To characterize the sequences mapping to the target reference genome as well as the ones mapping to the non-target and human references using two characteristics of ancient DNA: short fragment size [4, 46, 47] measured as median read length (mRL) and deamination patterns [48, 49] measured as post-mortem damage scores (PMD, [30]). For each sample, we define the PMD ratio (PMD^R^) as the fraction of sequences that display a PMD score > 5. Therefore, a higher PMD^R^ value indicates that the sample contains more sequences with larger PMD scores, thus it contains more ancient DNA sequences.

In order to estimate the amount of data lost using competitive mapping we identified conserved regions between the elephant and human genomes as well as the dog and human genomes. We first used a custom script to split the human reference genome into overlapping 30 bp long sequences with a step size of 1 bp. We then mapped the obtained short sequences to the other two reference genomes, dog and elephant, using *BWA* [50]. For each mapping, we filtered out reads with mapping quality below 30 and identified all genomic regions with at least one read mapped. The resulting BED files were used together with *samtools flagstat* to estimate the number of reads mapping to conserved regions before and after competitive mapping.

## Supplementary Information

**Additional file 1.** Supplementary Information which contains Extended results note 1, Figure S1 and Supplementary Table 1.

## Data Availability

All the data generated in the current study are available in the European Nucleotide Archive (ENA), accession number PRJEB41038.
