# Supplementary material for: Competitive mapping allows for the identification and exclusion of human DNA contamination in ancient faunal genomic datasets
Source: BMC Genomics. 2020 Nov 30;21:844. doi: 10.1186/s12864-020-07229-y (PMC7708127; doi:10.1186/s12864-020-07229-y)
Supplement: Supplementary file 1 — Additional file 1. Supplementary Information which contains Extended results note 1, Figure S1 and Supplementary Table 1. [file 12864_2020_7229_MOESM1_ESM.zip › Contamination_BMC_Genomics_R1_SI.docx]

**Supplementary Information for: “Competitive mapping allows for the identification and exclusion of human DNA contamination in ancient faunal genomic datasets”**

Tatiana R. Feuerborn, Elle Palkopoulou, Tom van der Valk, Johanna von Seth, Arielle R. Munters, Patrícia Pečnerová, Marianne Dehasque, Irene Ureña, Erik Ersmark, Vendela Kempe Lagerholm, Maja Krzewinska, Ricardo Rodríguez-Varela, Anders Götherström, Love Dalen, David Díez-del-Molino

Supplementary Information contains:

Extended results note 1

Figure S1

Supplementary Table 1

**Extended results note 1**

After competitive mapping, we analyzed the read length and post-mortem damage patterns at the species level and found that the mammoth samples displayed a clearer distinction in PMD^R^ than the dog samples when comparing the reads mapped to the target and to the human parts of the concatenated reference (Fig. 3A, 3D). This may be related to both the age of the samples post-mortem and the age since collection. PMD scores are roughly proportional to the sample’s age [[1]](https://paperpile.com/c/Ar4zLn/tzF1j), and while the mammoth samples are thousands of years old, they have been housed in collections for less than 30 years. The dog samples on the other hand were only a maximum of 1,000 years old (Supplementary Table 1) but were housed in museum collections since their excavation or collection for up to 125 years. Because the conditions in museum collections are usually far from ideal for DNA preservation [[2, 3]](https://paperpile.com/c/Ar4zLn/LFXS5+sKN4m), this extended period of storage could have had an impact on the preservation of both endogenous dog and contaminant human DNA sequences in the ancient dog samples in comparison to the mammoth samples.

[1. Skoglund P, Northoff BH, Shunkov MV, Derevianko AP, Pääbo S, Krause J, et al. Separating endogenous ancient DNA from modern day contamination in a Siberian Neandertal. Proc Natl Acad Sci U S A. 2014;111:2229–34.](http://paperpile.com/b/Ar4zLn/tzF1j)

[2. Burrell AS, Disotell TR, Bergey CM. The use of museum specimens with high-throughput DNA sequencers. J Hum Evol. 2015;79:35–44.](http://paperpile.com/b/Ar4zLn/LFXS5)

[3. Díez-del-Molino D, Sánchez-Barreiro F, Barnes I, Gilbert MTP, Dalén L. Quantifying Temporal Genomic Erosion in Endangered Species. Trends Ecol Evol. 2018;33:176–85.](http://paperpile.com/b/Ar4zLn/sKN4m)

**
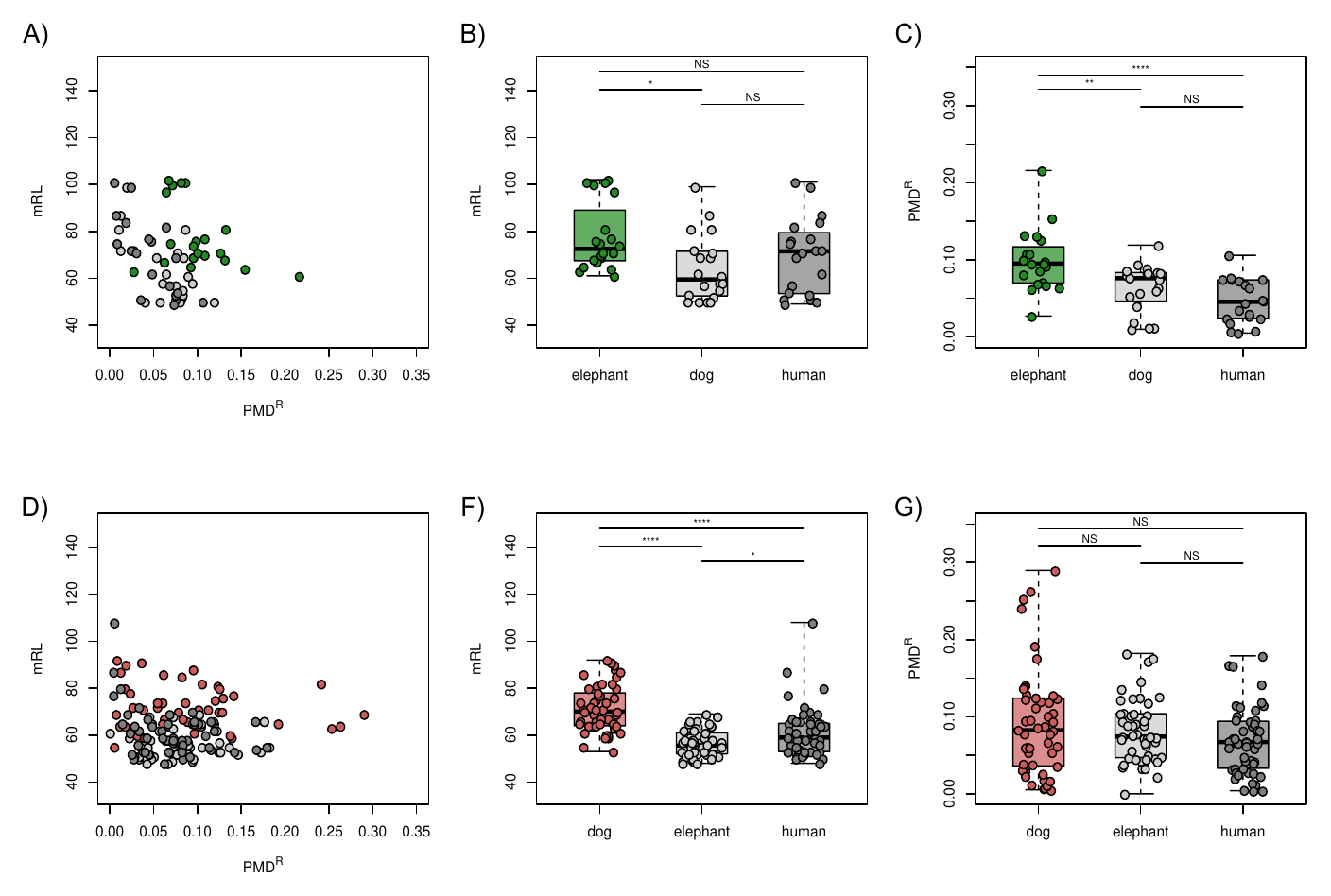
Figure S1: Characterization of sequences mapping to the target, non-target and human references.** A) Comparisons of PMD^R^ and mRL for all mammoth samples. B) mRL for mammoth sequences mapping to the elephant, dog and human references. C) PMD^R^ for mammoth sequences mapping to the elephant, dog and human references. D) Comparisons of PMD^R^ and mRL for all ancient dog samples. D) mRL for dog sequences mapping to the elephant, dog and human references. F) PMD^R^ for dog sequences mapping to the elephant, dog and human references. All pairwise comparisons are done using Tukey's tests. In all cases, NS: p-value >0.05, *: p-value <0.05, **: p-value < 0.01 and ****: p-value < 0.0001.

**Table S1: Sample descriptions and mapping statistics.** All mammoth samples are radiocarbon dated. Estimated ages are provided including the ^14^C error.
